# Tuning Immobilized Commercial Lipase Preparations Features by Simple Treatment with Metallic Phosphate Salts

**DOI:** 10.3390/molecules27144486

**Published:** 2022-07-13

**Authors:** José R. Guimarães, Diego Carballares, Paulo W. Tardioli, Javier Rocha-Martin, Roberto Fernandez-Lafuente

**Affiliations:** 1 Departamento de Biocatálisis, ICP-CSIC, Campus UAM-CSIC, 28049 Madrid, Spain; renatoge74@gmail.com (J.R.G.); diego.carballares@csic.es (D.C.); 2 Laboratory of Enzyme Technologies (LabEnz), Department of Chemical Engineering, Federal University of São Carlos (DEQ/UFSCar), Rod. Washington Luís, km 235, São Carlos 13565-905, Brazil; pwtardioli@ufscar.br; 3 Department of Biochemistry and Molecular Biology, Faculty of Biology, Complutense University of Madrid, José Antonio Novais 12, 28040 Madrid, Spain; 4 Center of Excellence in Bionanoscience Research, King Abdulaziz University, Jeddah 21589, Saudi Arabia

**Keywords:** solid phase enzyme mineralization, nanoflowers, immobilized lipases, enzyme specificity, enzyme stability

## Abstract

Four commercial immobilized lipases biocatalysts have been submitted to modifications with different metal (zinc, cobalt or copper) phosphates to check the effects of this modification on enzyme features. The lipase preparations were Lipozyme^®^TL (TLL-IM) (lipase from *Thermomyces lanuginose*), Lipozyme^®^435 (L435) (lipase B from *Candida antarctica*), Lipozyme^®^RM (RML-IM), and LipuraSelect (LS-IM) (both from lipase from *Rhizomucor miehei*). The modifications greatly altered enzyme specificity, increasing the activity versus some substrates (e.g., TLL-IM modified with zinc phosphate in hydrolysis of triacetin) while decreasing the activity versus other substrates (the same preparation in activity versus *R*- or *S*- methyl mandelate). Enantiospecificity was also drastically altered after these modifications, e.g., LS-IM increased the activity versus the *R* isomer while decreasing the activity versus the *S* isomer when treated with copper phosphate. Regarding the enzyme stability, it was significantly improved using octyl-agarose-lipases. Using all these commercial biocatalysts, no significant positive effects were found; in fact, a decrease in enzyme stability was usually detected. The results point towards the possibility of a battery of biocatalysts, including many different metal phosphates and immobilization protocols, being a good opportunity to tune enzyme features, increasing the possibilities of having biocatalysts that may be suitable for a specific process.

## 1. Introduction

Lipases are among the most utilized enzymes, both academically and industrially [1,2,3,4,5]. This is because they are very robust biocatalysts, able to perform a wide variety of reactions, such as hydrolysis, esterifications [6,7,8,9], transesterifications [10,11,12,13,14] or acidolysis [15,16]. They can be used in a wide variety of reaction media (aqueous medium, organic solvents [17,18], solvent-free [19], ionic liquid [20,21,22], deep eutectic solvents [23] and supercritical fluids [24,25,26,27]) and have a wide substrate specificity, accepting substrates that are very different structurally. However, this is in many instances bound to a high regioselectivity, enantiospecificity and selectivity [28,29,30,31,32,33,34].

In fact, they are one of the most successful examples of enzymes presenting promiscuous activities [1,35,36,37,38,39,40].

In homogeneous media, most lipases present a conformational equilibrium between a form where a polypeptide chain (lid) isolates its active center from the medium (closed form) and a form where this lid is shifted exposing the active center to the medium (open form) [41,42,43]. In the presence of insoluble drops of substrate, the open form of the lipase becomes adsorbed on the hydrophobic surface and becomes stabilized [42]. This can occur also on any other hydrophobic surface, such as the open form of other lipase molecule [44,45,46,47,48], a hydrophobic protein [49,50] or a hydrophobic matrix [51].

The use of immobilized enzymes permits enzyme recovery and their reuse, providing that the enzyme remains active [52,53,54,55,56,57,58,59]. This also enables a simpler control of the reaction and also the utilization of any reactor configuration [60]. Researchers have tried to couple this immobilization step in the design of industrial enzyme biocatalysts in a way to improve many enzyme features. That way, a proper immobilization may improve enzyme stability by different reasons (recently reviewed [61]), and in that way, increase the range of conditions where the enzyme may be utilized, increasing the prospect of success in the design of a bioprocess [62,63]. Furthermore, enzyme immobilization will alter enzyme selectivity, specificity and activity [28], may reduce inhibitions and, if adequately designed, enable the one step immobilization-purification of the target enzyme [64,65,66].

In the last decade, the production of hybrid enzyme nanoflowers proved to be an immobilization method able to improve some enzyme features, such as enzyme stability and activity [67,68,69,70,71,72,73,74]. In some cases, this technique has been applied to immobilize lipases [75,76,77,78,79,80,81,82,83,84,85,86,87,88,89].

However, the mechanical fragility of nanoflowers makes their use complex in most reactor configurations. One alternative to solve this problem in some instances is to trap the nanoflowers in solids with better mechanical performance or confer a magnetic character to the biocatalyst [67,76,78,86,90,91,92].

Recently, we tried to reproduce this strategy using immobilized enzymes [93]. We found that in some instances, the enzyme features (activity or stability) were significantly improved [93]. Although there was no evidence of the production of hybrid enzyme-lipase nanoflower structures, the modification of the enzyme nucleation sites with the metal phosphate was assumed to be the cause of these positive effects.

In this new research, we investigate if this simple immobilized enzyme treatment may improve some of the most utilized commercial immobilized lipase preparations. The lipase B from *Candida antarctica* immobilized on moderately hydrophobic Lewatit VP OC 1600 via interfacial activation [94], with commercial name Novozym^®^ 435 [94], has been one of the used preparations. Additionally, the lipase from *Rhizomucor miehei* immobilized on Duolite ES 562, a weak anion-exchange resin based on phenol-formaldehyde copolymers (RML-IM), has been included in this study [95] and a new biocatalyst called LipuraSelect, with scarce information available on the preparation way (that is, the immobilization mechanism is unknown). Finally, the lipase from *Thermomyces lanuginosus* immobilized on a cationic silicate (TLL-IM) was included [96]. All these commercial preparations have been treated with phosphate and the chloride salts of Cu^2+^, Co^2+^ and Zn^2+^, and their functional properties have been analyzed.

## 2. Results and Discussion

### 2.1. Modification of Commercial Immobilized TLL (IM-TL)

IM-TL was modified as indicated in methods and the activities of the different biocatalysts versus diverse substrates were determined (Table 1). The modification with phosphate and Co^2+^ produced a significant decrease of the activity of IM-TL versus triacetin, almost by 50%. However, the activity significantly increased using Cu^2+^ (by a 40%) and even more significantly using Zn^2+^ (almost a 70%). However, all treatments produced a decrease in the activity versus both methyl mandelate isomers, more significant for the S-isomer. This means that the treatment alters both specificity versus the substrates and enantiospecificity. In the most significant cases, the activity ratio of activities between *R*-methyl mandelate and triacetin (which is initially just under 1.3) increases to 2 when the enzyme is modified using phosphate and Co^2+^, or to 0.6 using phosphate and Zn^2+^. The enantiospecificity for the isomers of methyl mandelate also changes, but not so significantly from the initial ratio of reaction rate using *R*/*S* isomer of almost 1.6, to almost 2 if the biocatalyst is modified with phosphate and Co^2+^. These changes suggested that the metal phosphate modification should produce quite large changes in the functional properties of the enzyme (very likely caused by enzyme conformational changes), as reported in many papers regarding the effects of the immobilization protocol [97,98,99,100,101,102,103,104,105,106] or the chemical or physical modification of the enzymes [107,108,109,110,111,112,113] on enzyme specificity or selectivity. The fact that an enzyme is immobilized did not mean that the enzyme mobility is fully suppressed, and chemical or physical modifications can induce conformational changes. For example, it has been shown that the blocking of TLL-octyl-vinyl sulfone biocatalyst with different reagents can fully alter the enzyme functionality as well as the enzyme structure [114].

**Figure 1:**
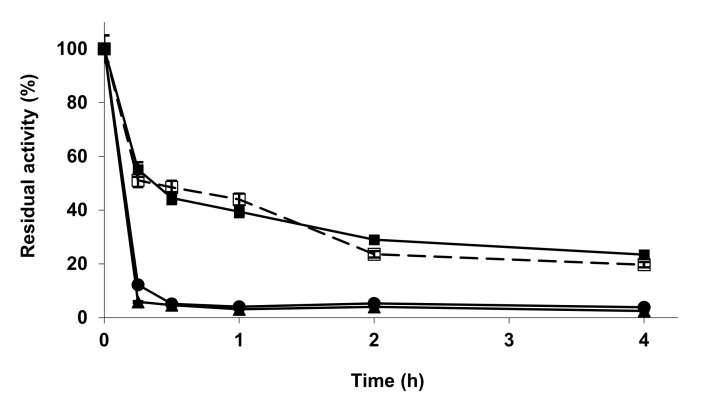
Inactivation courses of TLL-IM unmodified and modified with metallic salt/sodium phosphate. The inactivation was performed with 10 mM Tris buffer at pH 7.0 and 75 °C. Other specifications are described in the Methods section. Unmodified TLL-IM (open squares and dotted line); TLL-IM modified with ZnCl_2_/sodium phosphate (solid squares); CuCl_2_/sodium phosphate (solid circles); CoCl_2_/sodium phosphate (solid triangles).

Figure 1 shows the inactivation course of the different IM-TL biocatalysts. While Co^2+^/Cu^2+^ and phosphate treatment produced a drastic decrease in enzyme stability, the treatment with Zn^2+^ resulted in a biocatalyst that fully maintained the enzyme stability at pH 7. These results disagree with the results obtained using octyl-agarose-TLL [93], where stability was greatly improved after this treatment, while the activity (versus *p*-NPB) was slightly decreased. This suggested that the immobilization protocol could greatly alter the effect of the metal phosphate modification of the biocatalyst. This result agrees with previous reports that state that the immobilization protocol can alter the effect of the enzyme modification on enzyme features, either chemical or physical [112,113]. In the case of IM-TLL, the modification with phosphate and Zn^2+^ was permitted to increase the enzyme activity versus some substrates, altering enzyme specificity, while maintaining enzyme stability.

### 2.2. Modification of Commercial Immobilized CALB (L435)

Table 2 shows the effect of the modification of L435 with different metal phosphate on the enzyme activities versus different substrates. Using triacetin, Zn^2+^ and phosphate treatment produced a 25% increase of enzyme activity, while the other two salts have a marginal negative effect. Using both isomers of methyl mandelate, the decrease in activity was more significant, to 1/3 using phosphate and Co^2+^ for the *R* isomer, and 1/5 using the *R* isomer. This produced a great effect on enzyme specificity, while the activity versus triacetin/activity versus *R* isomer of the initial biocatalyst was 2.9 for the unmodified enzyme, this increased to 6.5 for the enzyme modified using Zn^2+^ and phosphate, 5.9 using Cu^2+^ or 8.5 using Co^2+^. Regarding the activity versus *R*/activity versus *S* isomers, they are in the range of 1.3–1.4 for the initial preparation or the biocatalysts treated with zinc phosphate, 1.9 if treated with copper phosphate and over 2 if treated using cobalt phosphate.

**Figure 2:**
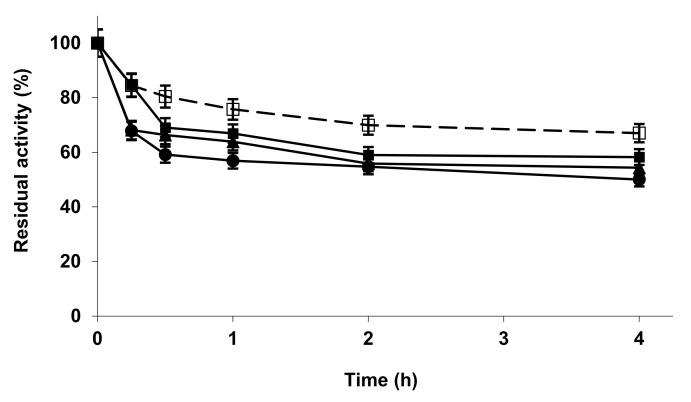
Inactivation courses of L435 unmodified and modified with metallic salt/sodium phosphate. The inactivation was performed with 10 mM Tris buffer at pH 7.0 and 75 °C. Other specifications are described in the Methods section. Unmodified L435 (open squares and dotted line); L435 modified with ZnCl_2_/sodium phosphate (solid squares); CuCl_2_/sodium phosphate (solid circles); CoCl_2_/sodium phosphate (solid triangles).

Figure 2 shows the inactivation courses, and it becomes obvious that all the modifications presented a similarly small negative effect on enzyme stability at pH 7. Using octyl-agarose-CALB, the results were again quite different, with some increase on enzyme stability and activity. Explanations for these results may be similar to those given in the case of TLL.

### 2.3. Modification of Commercial Immobilized RML (RM-IM and LS-IM)

In the case of RML, we have got two different commercial preparations, RM-IM and LS-IM. The activities of both biocatalysts (intact and metal phosphate modified) may be found in Table 3 (RM-IM) and Table 4 (LS-IM). First, we will compare the activities of both biocatalysts versus the different substrates used in this study. RM-IM was slightly more active versus triacetin than LS-IM; however, it was significantly more active (almost by a 10-fold factor) versus *R*-methyl mandelate. The hydrolysis of the *R* isomer was more rapid using RM-IM (1.15-fold) than using LS-IM, while LS-IM preferred the *S*-isomer (1.4-fold). That way, both RML biocatalysts presented a very different specificity and enantiospecificity, as has been reported in many other instances for RML immobilized on different supports [97,98,99,100,101,102].

When RM-IM was treated with the metal salts, the activity versus triacetin decreased much more than the activity versus the methyl mandelate esters. The activity versus triacetin decreased to 81% when the biocatalyst was modified with zinc or cobalt phosphates, but below 50% if using copper phosphate. The activity versus *R*-methyl mandelate decreased to around 10% in the more drastic case (when modified with zinc phosphate), while using the *S*-isomer the activity was maintained. That way, the activity versus triacetin/activity versus *R* methyl mandelate ratio was moved from 7.6 for the original biocatalyst to 3.7 for the enzyme modified with copper phosphate. The changes in the *R*/*S* methyl mandelate activity ratio were much smaller (from the original 1.15 to almost 1).

Results were very different using LS-IM. Activity versus triacetin decreased after the treatment, but it decreased more using zinc (below 70%), while the other two preparations maintained around 80% of the initial activity. In the case of methyl mandelate, the effects were very different using each of the isomers. The activity versus *R* methyl mandelate increased by 60% when treated with copper and by 20% using cobalt, while the use of zinc salts decreased the activity by 20%. However, the activity versus the *R* isomer decreased in all cases, more using zinc (to 50%) and less using cobalt (75%). This means some changes in enzyme specificity, going from an activity of 62 versus triacetin/*S* methyl mandelate (much higher than using RM-IM) to 82 (after modification with Zn), and more significant changes in the activity versus *S/R* methyl mandelate ratio. The unmodified biocatalyst presented an activity ratio of 1.4. All modified biocatalysts preferred the *R* isomer, giving a value around 0.6 when modified with copper. That way, for this preparation, the modification produced a more significant change in the enantiospecificity than in the specificity, in opposition with RM-IM.

The comparison between two biocatalysts of the same enzyme confirms that the effect of the modification with metal phosphate is greatly dependent on the immobilization protocol, as has been reported for other physical or chemical immobilized enzyme modifications [107,110,111,112,113,115].

**Figure 3:**
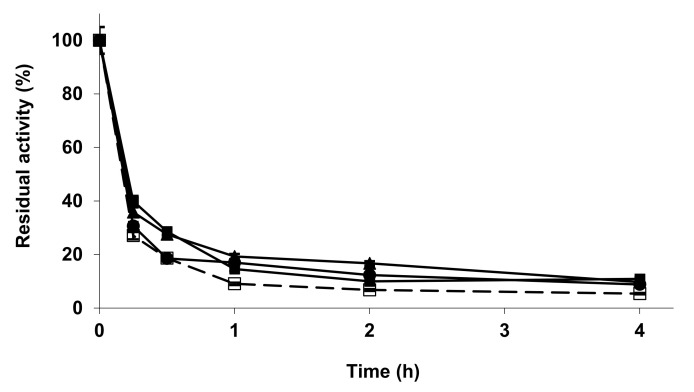
Inactivation courses of RM-IM unmodified and modified with metallic salt/sodium phosphate. The inactivation was performed with 10 mM Tris buffer at pH 7.0 and 60 °C. Other specifications are described in the Methods section. Unmodified RM-IM (open squares and dotted line); RM-IM modified with ZnCl_2_/sodium phosphate (solid squares); CuCl_2_/sodium phosphate (solid circles); CoCl_2_/sodium phosphate (solid triangles).

**Figure 4:**
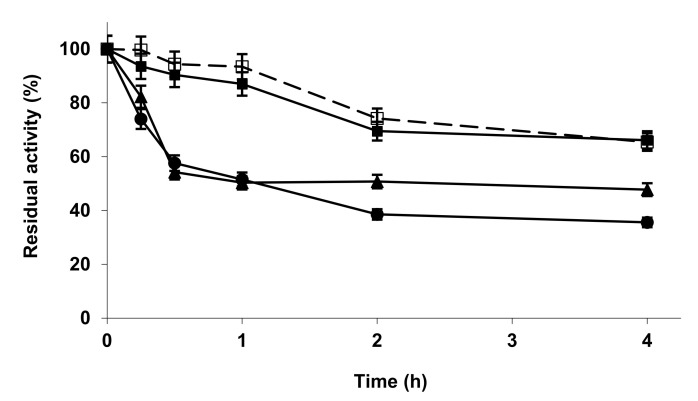
Inactivation courses of LS-IM unmodified and modified with metallic salt/sodium phosphate. The inactivation was performed with 10 mM Tris buffer at pH 7.0 and 60 °C. Other specifications are described in the Methods section. Unmodified LS-IM (open squares and dotted line); LS-IM modified with ZnCl_2_/sodium phosphate (solid squares); CuCl_2_/sodium phosphate (solid circles); CoCl_2_/sodium phosphate (solid triangles).

Figure 3 shows the effect of the metal phosphate treatments on the stability of RM-IM. All the treatments had a scarce, but positive effect on enzyme stability. The comparison with Figure 4 shows that LS-IM is much more stable than RM-IM. The modification of LS-IM with zinc phosphate had no effect on enzyme stability, while the other two treatments produced a clear decrease on enzyme stability. Again, different enzyme preparations of the same enzyme exhibited a very different response to the treatment with metal phosphate.

## 3. Materials and Methods

### 3.1. Materials

In this study, we have employed different commercial immobilized lipase. Lipozyme^®^TL (TLL-IM), Lipozyme^®^435 (L435), Lipozyme^®^RM (RML-IM) and LipuraSelect (LS-IM) were kindly donated by Novozymes Spain (Madrid, Spain). Triacetin, (*R*)- and (*S*)-methyl mandelate, zinc chloride (ZnCl_2_), copper chloride (CuCl_2_), cobalt chloride (CoCl_2_), sodium chloride (NaCl) and acetonitrile for HPLC (gradient grade, ≥99.9%) were purchased from Sigma-Aldrich (St. Louis, MO, USA). All other reagents were of analytical grade.

### 3.2. Methods

#### 3.2.1. Modification of Immobilized Enzyme with Metallic Salt/Phosphate

TLL-IM, L435, RML-IM and LS-IM were modified with metallic salt/phosphate following the procedure described by Guimarães et al. [93]. A mass of 1 g of immobilized enzyme was suspended in 10 mL of saline buffer (10 mM sodium phosphate buffer and 125 mM NaCl) at pH 7.4 and, then, 400 µL of 230 mM of the corresponding metal salt was added. The enzyme treatment was conducted at room temperature under gentle stirring for 5 h in an orbital shaker at 550 rpm. After modification, the suspension was filtered and the biocatalysts were washed with distilled water (10 times with 10 volumes of water), and stored at 4 °C.

#### 3.2.2. Thermal Inactivation of the Different Lipase Preparations

In a standard experiment, 1 g of immobilized biocatalyst was suspended in 10 mL of 10 mM Tris-HCl at pH 7.0 and incubated at different temperatures. Phosphate was discarded as medium to inactivate the immobilized enzymes, as it has been reported to be negative for enzyme stability. The low buffer concentration prevents risks of enzyme release for the biocatalysts based on ion exchange [116,117].

The temperatures were selected to ensure reliable but not too long half-lives of the unmodified immobilized enzymes (TLL-IM and L435: 75 °C; RML-IM and LS-IM: 60 °C). Periodically, samples of 0.3 mL of inactivation suspension were collected after homogenization using a pipette with a cut tip to determine their residual activities. Residual activities were defined as activity of the biocatalyst after the indicated inactivation time divided by its initial and expressed in percentage. The experiments were performed, employing triacetin as substrate for the immobilized biocatalyst.

#### 3.2.3. Enzyme Activity Assays

One unit of activity (U) was defined as the amount of enzyme that hydrolyzes one µmol of substrate per minute under the described conditions.

##### Hydrolysis of Triacetin

A volume of 0.3 mL of immobilized enzyme suspension (166 mg/mL) was added to 3 mL of 50 mM of triacetin prepared in 50 mM of sodium phosphate buffer at pH 7.0. Hydrolysis was carried out at 25 °C under magnetic stirring (100 rpm). The hydrolytic activity in triacetin was quantified by detection of 1,2 and 1,3 diacetin (under these conditions, 1,2 diacetin suffers acyl migration, giving 1,3 diacetin) [118]. The degree of conversion was calculated by HPLC in a Waters 486 chromatograph (Waters, Millford, UK.) equipped with a UV/VIS detector (set to 230 nm) [118] using a Kromasil C18 column (15 cm × 0.46 cm) with a mobile phase composed of 15% (*v*/*v*) of water and 85% (*v*/*v*) of acetonitrile with a flow rate of 1 mL/min. The retention times were 4 min for 1,2 and 1,3 diacetins (under these conditions eluted at the same time) and 18 min for triacetin. Conversions of 15–20% were used to calculate enzyme activity [119].

##### Hydrolysis of *R*- or *S*-Methyl Mandelate

A mass of 0.05 g of commercial immobilized lipase were added to 3 mL of 50 mM *R*- or *S*-methyl mandelate in 50 mM sodium phosphate buffer solution at pH 7.0. Hydrolysis was carried out at 25 °C under magnetic stirring (100 rpm). The substrate and product concentrations were determined by HPLC using a Waters 486 chromatograph (Waters, Millford, UK) equipped with a UV/VIS detector (set to 230 nm) [114] using a Kromasil C18 column (15 cm × 0.46 cm) with a mobile phase composed of 10 mM ammonium acetate and acetonitrile (35–65% (*v*/*v*)) at pH 2.8 with a flow rate of 1 mL/min. The retention times were 2.5 min for mandelic acid and 4.2 min for the *R*- or *S*-methyl mandelate. Conversions of 15–25% were used to calculate enzyme activity [120].

## 4. Conclusions

The modification of commercial preparations of immobilized lipases with metal phosphate, in a treatment similar to the production of nanoflowers, alters enzyme specificity. These changes means that while the enzyme activity may increase for some substrates, it may decrease for some others. This also fully alters enzyme enantiospecificity. The best treatment is different for each enzyme, substrate and even for each immobilization protocol. The results in this paper point that the immobilization protocol can play a critical role on the effects of the treatment. Regarding the stability, a feature that was reported as much improved using octyl-agarose-lipase, using the commercial preparations show a moderate impact, usually even decreasing enzyme stability, or producing marginal stabilization (in the case of RM-IM). Although in this paper, we have focused on the effects of the modification on the functional properties of the immobilized enzymes, it seems very interesting to further investigate the mechanism of this modification. The fact that the metal crystals also grew on the naked supports makes this a very complex goal.

From the results of this paper, it is proposed that the modification of a biocatalyst with a battery of metals (not reducing the study to the ones used in this paper) may open new opportunities for tailoring the enzyme features, increasing the opportunities to find a biocatalyst with the optimal properties for a specific process.

## Figures and Tables

**Table 1:** Specific activity of different biocatalysts with 50 mM *R*- or *S*-methyl mandelate (pH 7, 25 °C) and 50 mM of triacetin (pH 7, 25 °C). Experiments were performed as described in the Methods section.

Biocatalysts	Activity (U/g)		
	Triacetin	*R* Mandelate	*S* Mandelate
TLL-IM	7.73 ± 0.35	9.90 ± 0.49	6.33 ± 0.57
TLL-IM-ZnP	13.02 ± 0.64	7.88 ± 0.27	5.31 ± 0.18
TLL-IM-CuP	10.80 ± 0.44	8.77 ± 0.37	5.12 ± 0.26
TLL-IM-CoP	4.08 ± 0.19	8.09 ± 0.40	4.18 ± 0.32

**Table 2:** Specific activity of different biocatalysts with 50 mM *R*- or *S*-methyl mandelate (pH 7, 25 °C) and 50 mM of triacetin (pH 7, 25 °C). Experiments were performed as described in the Methods section.

Biocatalysts	Activity (U/g)		
	Triacetin	*R* Mandelate	*S* Mandelate
L435	119.0 ± 5.2	42.6 ± 1.8	31.2 ± 1.6
L435-ZnP	149.9 ± 7.8	23.1 ± 0.9	17.3 ± 0.9
L435-CuP	113.9 ± 5.9	19.4 ± 1.0	11.5 ± 0.6
L435-CoP	116.2 ± 6.9	13.3 ± 0.6	6.4 ± 0.4

**Table 3:** Specific activity of different biocatalysts with 50 mM *R*- or *S*-methyl mandelate and 50 mM of triacetin (pH 7, 25 °C). Experiments were performed as described in the Methods section.

Biocatalysts	Activity (U/g)		
	Triacetin	*R* Mandelate	*S* Mandelate
RML-IM	86.2 ± 4.7	11.3 ± 0.8	9.8 ± 0.5
RML-IM-ZnP	70.00 ± 3.7	9.9 ± 0.6	9.8 ± 0.4
RML-IM-CuP	38.4 ± 1.9	10.2 ± 0.6	9.8 ± 0.4
RML-IM-CoP	69.9 ± 3.8	10.1 ± 0.5	9.9 ± 0.2

**Table 4:** Specific activity of different biocatalysts with 50 mM *R*- or *S*-methyl mandelate and 50 mM of triacetin (pH 7, 25 °C). Experiments were performed as described in the Methods section.

Biocatalysts	Activity (U/g)		
	Triacetin	*R* Mandelate	*S* Mandelate
LS-IM	80.5 ± 4.9	0.92 ± 0.04	1.29 ± 0.06
LS-IM-ZnP	54.9 ± 3.0	0.73 ± 0.04	0.67 ± 0.03
LS-IM-CuP	63.8 ± 3.3	1.50 ± 0.09	0.88 ± 0.06
LS-IM-CoP	63.7 ± 3.9	1.10 ± 0.07	0.99 ± 0.05

## Data Availability

Not applicable.

## Sample Availability

Samples are available from the authors.
